# Gasdermin D mediates the pathogenesis of neonatal-onset multisystem inflammatory disease in mice

**DOI:** 10.1371/journal.pbio.3000047

**Published:** 2018-11-02

**Authors:** Jianqiu Xiao, Chun Wang, Juo-Chin Yao, Yael Alippe, Canxin Xu, Dustin Kress, Roberto Civitelli, Yousef Abu-Amer, Thirumala-Devi Kanneganti, Daniel C. Link, Gabriel Mbalaviele

**Affiliations:** 1 Division of Bone and Mineral Diseases, Washington University School of Medicine, St. Louis, Missouri, United States of America; 2 Division of Oncology, Washington University School of Medicine, St. Louis, Missouri, United States of America; 3 Confluence Discovery Technologies, Inc., St. Louis, Missouri, United States of America; 4 Department of Orthopedic Surgery, Washington University School of Medicine and Shriners Hospital for Children, St. Louis, Missouri, United States of America; 5 Department of Immunology, St. Jude Children’s Research Hospital, Memphis, Tennessee, United States of America; National Cancer Institute, UNITED STATES

## Abstract

Mutated NLRP3 assembles a hyperactive inflammasome, which causes excessive secretion of interleukin (IL)-1β and IL-18 and, ultimately, a spectrum of autoinflammatory disorders known as cryopyrinopathies of which neonatal-onset multisystem inflammatory disease (NOMID) is the most severe phenotype. NOMID mice phenocopy several features of the human disease as they develop severe systemic inflammation driven by IL-1β and IL-18 overproduction associated with damage to multiple organs, including spleen, skin, liver, and skeleton. Secretion of IL-1β and IL-18 requires gasdermin D (GSDMD), which—upon activation by the inflammasomes—translocates to the plasma membrane where it forms pores through which these cytokines are released. However, excessive pore formation resulting from sustained activation of GSDMD compromises membrane integrity and ultimately causes a pro-inflammatory form of cell death, termed pyroptosis. In this study, we first established a strong correlation between NLRP3 inflammasome activation and GSDMD processing and pyroptosis in vitro. Next, we used NOMID mice to determine the extent to which GSDMD-driven pyroptosis influences the pathogenesis of this disorder. Remarkably, all NOMID-associated inflammatory symptoms are prevented upon ablation of GSDMD. Thus, GSDMD-dependent actions are required for the pathogenesis of NOMID in mice.

## Abstract 2

Pyroptosis mediated by the pore-forming protein gasdermin D plays a crucial role in the pathogenesis of neonatal-onset multisystem inflammatory disease, a severe genetic autoinflammatory disorder resulting from activating mutations in the NLRP3/cryopyrin gene.

## Author summary

The NLRP3 inflammasome plays an important role in the maturation of interleukin (IL)-1β and IL-18. Accordingly, *NLRP3* gain-of-function mutations, which cause a spectrum of autoinflammatory disorders known as cryopyrin-associated periodic syndromes (CAPS), are associated with excessive IL-1β and IL-18 production. Although CAPS-associated inflammatory symptoms are treated with IL-1-blocking agents, emerging evidence indicates that some CAPS patients only partially respond to these drugs. Persistent inflammatory responses have also been reported in CAPS mice deficient in IL-1β and IL-18 signaling and may be the consequences of the pro-inflammatory cell death, pyroptosis, which is induced by gasdermin D (GSDMD), the other effector of the inflammasomes. Consistent with this view, we found that damage to multiple organs that manifested in a mouse model of CAPS was prevented by ablation of GSDMD.

## Introduction

NLRP3, also called cryopyrin, assembles an inflammasome complex upon sensing danger signals triggered by structurally different exogenous and endogenous molecular entities [1–3]. Failure to clear the insults or restore homeostasis leads to chronic activation of this inflammasome, a response that underlies various inflammatory and metabolic diseases, including gout, diabetes, and atherosclerosis [4]. Activating mutations in the *NLRP3* gene also cause constitutive activation of the NLRP3 inflammasome in patients with a spectrum of autoinflammatory disorders known as cryopyrinopathies or cryopyrin-associated periodic syndromes (CAPS), which include neonatal-onset multisystem inflammatory disease (NOMID), Muckle-Wells syndrome (MWS), and familial cold autoinflammatory syndrome (FCAS) [5, 6]. CAPS are monogenic disorders with some degree of genotype-phenotype correlation, with NOMID exhibiting the most severe manifestations [5, 6]. Each of the CAPS phenotypes displays multiple symptoms, including systemic inflammation, recurrent or chronic fever, and urticaria-like rash [5, 6].

Consistent with the NLRP3 inflammasome role in interleukin (IL)-1β and IL-18 maturation, cryopyrinopathies are associated with excessive production of these cytokines. Accordingly, IL-1-blocking drugs are widely used in the management of these disorders. However, it appears that some CAPS patients only partially respond to IL-1 biologics [7–9]. In addition, skeletal lesions, the hallmark of NOMID, are refractory to IL-1 blockade [10–13]. These clinical observations underscore the complexity of cryopyrinopathies by suggesting that other actions of the inflammasomes beyond maturation of cytokines also contribute to the pathogenesis of these disorders. Indeed, the NLRP3 inflammasome also processes gasdermin D (GSDMD) into GSDMD-N (N-terminal domain) and GSDMD-C (C-terminal domain) [14–16]. GSDMD-N translocates to the plasma membrane, where it binds phospholipids and forms pores at the plasma membrane through which IL-1β and IL-18 are secreted by living cells [17–19]. Sustained activity of the inflammasomes causes excessive maturation of GSDMD and pore formation; this leads to membrane perforation and, ultimately, pyroptosis [17, 20–23]. This form of cell death provokes the uncontrolled release of not only IL-1β and IL-18 but also cytoplasmic contents, resulting in the recruitment of immune cells and propagation of inflammation [17, 24]. Thus, pyroptosis is not a silent endpoint, but the extent to which this pathologic process influences the pathogenesis of cryopyrinopathies is unknown.

Knockin mice harboring specific mutations found in CAPS patients were engineered in an attempt to generate preclinical disease-relevant models for genotype-phenotype relationship studies [25–28]. These models recapitulate some clinical features though disease manifestations are, in general, more severe in mice than in humans. Nonetheless, these seminal studies revealed that pyroptosis may be responsible for the persistent inflammatory responses in mice with impaired IL-1β and IL-18 signaling [8, 29]. Here, we used NOMID mice to determine the role that GSDMD and pyroptosis play in this disease model. NOMID mice exhibited systemic inflammation, stunt growth, and damage to multiple organs. These anomalies were absent in NOMID mice lacking GSDMD, which were indistinguishable from wild-type (WT) littermates. These results reveal a nonredundant function of GSDMD in the onset and progression of NOMID in mice.

## Results and discussion

### Maturation of GSDMD and IL-1β, and pyroptosis, occur constitutively in NOMID cells

**Fig 1:**
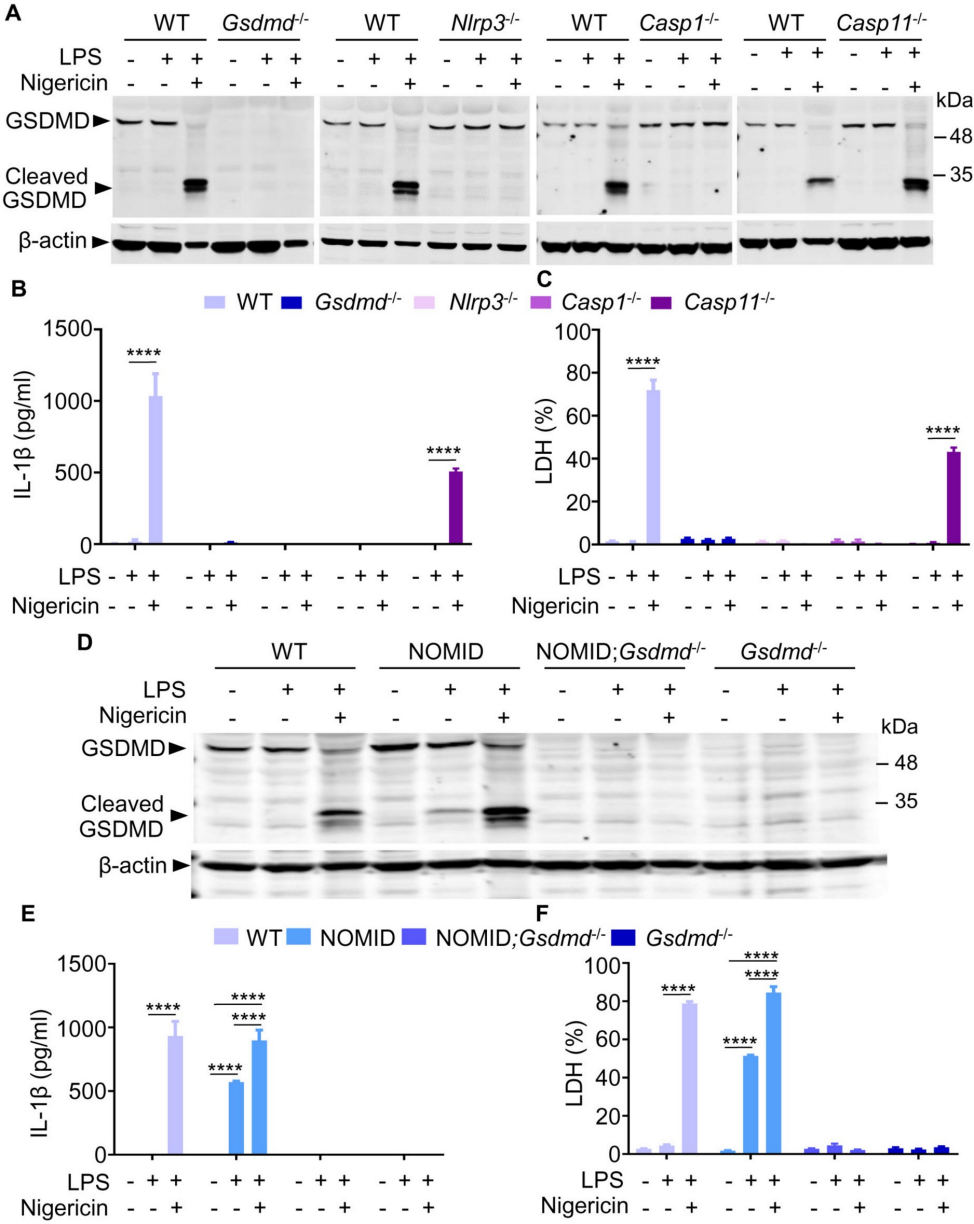
GSDMD cleavage correlates with IL-1β secretion and pyroptosis. BMMs were isolated from WT, *Gsdmd*^−/−^, *Nlrp3*^−/−^, *Casp1*^−/−^, *Casp11*^−/−^, NOMID, or NOMID;*Gsdmd*^−/−^ mice and were expanded in vitro in the presence of M-CSF-containing media. BMMs were primed with LPS for 3 hours and treated with nigericin for 30 minutes. (A, D) Western blot analysis of cell lysates. (B, E) IL-1β levels in conditioned media. (C, F) Percent of LDH release in conditioned media. Data are mean ± SEM from experimental triplicates and are representative of at least three independent experiments. The numerical values underlying Fig 1B, 1C, 1E and 1F can be found in S1 Data. ****P < 0.0001. BMM, bone marrow–derived macrophage; Casp, caspase; GSDMD, gasdermin D; IL-1β, interleukin-1β; LDH, lactate dehydrogenase; LPS, lipopolysaccharide; M-CSF, macrophage colony-stimulating factor; NOMID, neonatal-onset multisystem inflammatory disease; WT, wild-type.

The NLRP3 inflammasome complex—which comprises NLRP3 itself, the adapter protein, apoptosis-associated speck-like protein containing a CARD (ASC), and caspase-1—processes pro-IL-1β and pro-IL-18 into IL-1β and IL-18, respectively [1]. This inflammasome also cleaves GSDMD into GSDMD-N and GSDMD-C [14–16]. GSDMD-N forms pores at the plasma membranes through which IL-1β and IL-18 are secreted by living cells; excessive pore formation causes pyroptosis, a response that can be assessed in vitro by quantifying the release of lactate dehydrogenase (LDH) [18, 19]. Consistent with the literature, GSDMD was cleaved upon stimulation of WT mouse bone marrow–derived macrophages (BMMs) with lipopolysaccharide (LPS) and nigericin (Fig 1A). Two cleaved GSDMD fragments were detected; whether the larger fragment was further processed to generate the smaller fragment is not known. GSDMD maturation correlated with the release of not only IL-1β (Fig 1B and S1 Data) but also LDH (Fig 1C and S1 Data), indicating that BMMs undergo NLRP3 inflammasome-dependent pyroptosis under these experimental conditions. To reinforce this conclusion, GSDMD processing, cytokine production, and pyroptosis were determined using cells isolated from mice lacking GSDMD or components of either the NLRP3 canonical inflammasome (e.g., NLRP3 or caspase-1) or noncanonical inflammasome (e.g., caspase-11). Maturation of IL-1β and GSDMD was impaired in BMMs lacking any component of the classical NLRP3 inflammasome but was unaffected in *Casp11* null cells (Fig 1A–1C), as expected. These results strengthen the view that GSDMD is a key effector of the NLRP3 inflammasome pathway.

The identification of more than 100 NLRP3 sequence variants underscores the challenges of genotype–phenotype relationship studies for CAPS [30]. In efforts to fill this gap, several preclinical CAPS-relevant models were developed [25–28]. They included knockin mice, which harbored a D301N NLRP3 mutation, the mouse ortholog of the human D303N mutation found in NOMID patients [25]. Mating of *Nlrp3*^*fl(D301N)/+*^ mice with *lysozyme M-Cre*^*−/+*^ (*LysM-Cre*^*−/+*^) mice yielded control and *Nlrp3*^*fl(D301N)/+*^;*LysM-Cre*^*−/+*^ mice, in which the autosomal dominant mutation in *Nlrp3* was induced in myeloid cells; these mice are referred to as NOMID mice. We previously reported that the phenotype of NOMID mice with myeloid-restricted activation of NLRP3, which included systemic inflammation and skeletal anomalies, resembled that of mice broadly expressing the mutated protein [25, 31, 32]. This mouse model provided the opportunity to determine the impact of GSDMD deficiency in the pathogenesis of NOMID. Consistently, GSDMD cleavage in WT BMMs required priming and secondary signals triggered by LPS and nigericin, respectively (Fig 1D). By contrast, GSDMD proteolysis in NOMID BMMs was induced by LPS alone though the response was maximal in the presence of the ionophore. Likewise, secretion of IL-1β and LDH by WT cells necessitated the combined actions of LPS and nigericin, whereas these responses were significantly induced by the endotoxin alone in NOMID cells (Fig 1E and 1F; and S1 Data). Notably, secretion of IL-1β and LDH was abolished in cells lacking GSDMD. Thus, mature IL-1β is constitutively produced in NOMID cells, but its release requires GSDMD.

### Anomalies in NOMID mice are prevented by deletion of *Gsdmd*

**Fig 2:**
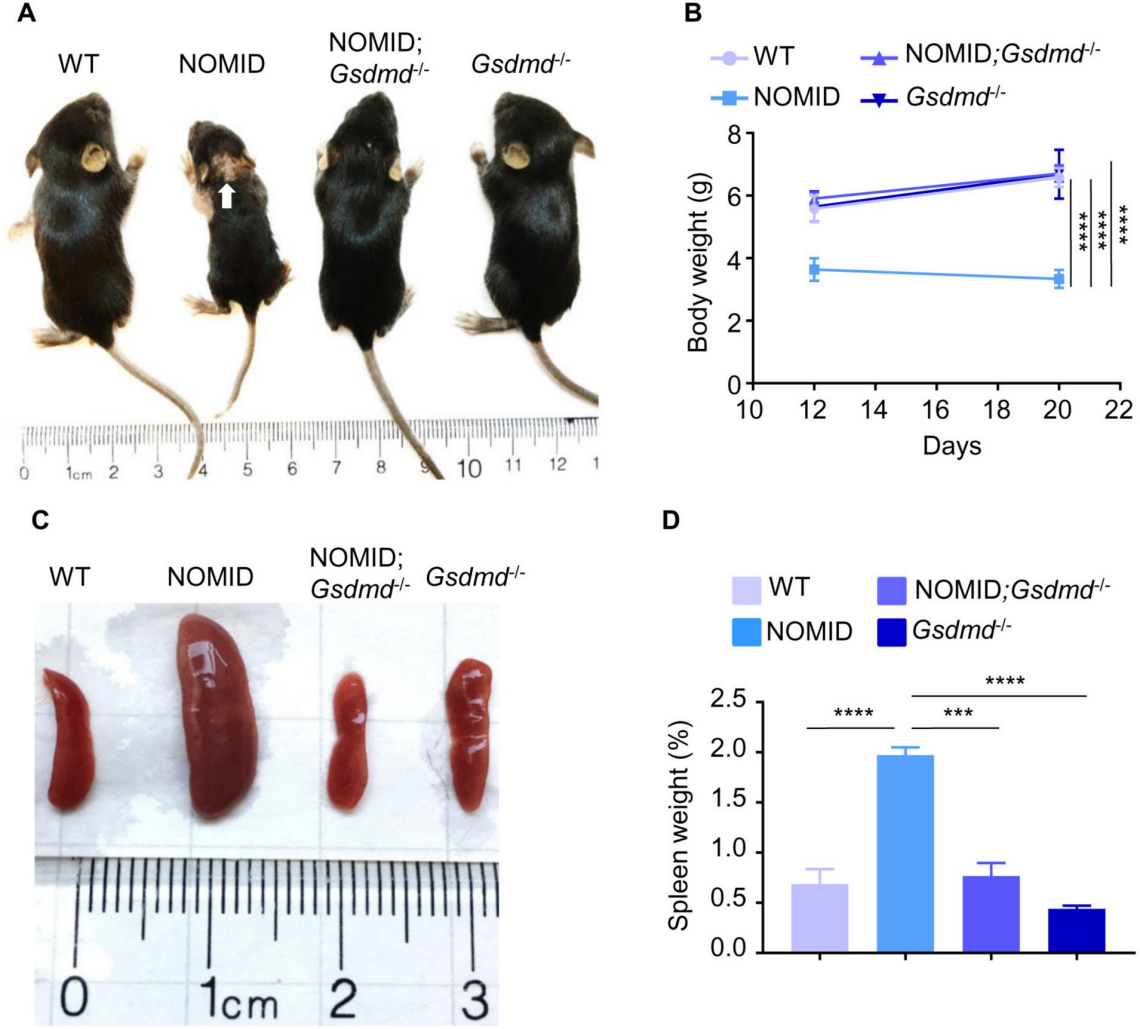
GSDMD deficiency prevents the stunt and splenomegaly phenotype of NOMID mice. (A) Representative pictures of mice from each genotype. White arrow indicates skin lesions. (B) Body weight of mice. (C) Representative pictures of the spleen of mice from each genotype. (D) Percent of spleen weight. Data are mean ± SEM from WT mice (3 males and 1 female), NOMID mice (3 males and 2 females), NOMID;*Gsdmd*^*−/−*^ mice (2 males and 1 female), and *Gsdmd*^*−/−*^ mice (3 males and 4 females). The weight of 1 male NOMID;*Gsdmd*^*−/−*^ mouse who was left to age is 5 g on day 12, 5.4 g on day 21, and 20.7 g on day 66. The numerical values underlying Fig 2B and 2D can be found in S1 Data. ***P < 0.0005; ****P < 0.0001. GSDMD, gasdermin D; NOMID, neonatal-onset multisystem inflammatory disease; WT, wild-type.

NOMID mice are runted, and they usually die by 2 to 3 weeks of age [25, 31], whereas *Gsdmd* null mice are apparently normal [16]. Consistent with these reports, NOMID pups were indistinguishable from WT and *Gsdmd* null littermates at birth but exhibited growth retardation and significantly lower body weight by 12 days of age (Fig 2A and 2B; S1A Fig and S1 Data). Additional macroscopic aberrations in NOMID mice included the presence of skin lesions (Fig 2A) and splenomegaly (Fig 2C and 2D; S1 Data). Skin and spleen abnormalities and the small body size phenotype of NOMID were all normalized in mutant mice lacking GSDMD (Fig 2A–2D). Growth delay, systemic inflammation, perinatal lethality, and spleen and skin abnormalities have been reported for other models of CAPS [26, 27, 29]. Deletion of *Il-1* receptor completely abolished these outcomes in NOMID mice but not in FCAS and MWS mice [8, 29], findings that are consistent with the view that, in contrast to humans, FCAS and MWS are unexpectedly more severe than NOMID in mice. The release of not only IL-1β and IL-18 but also other pro-inflammatory factors during pyroptosis may be responsible for the persistent residual inflammatory responses in FCAS and MWS models. Thus, it will be informative to determine the effects of GSDMD deficiency on disease progression in other preclinical models of CAPS.

While we were wrapping up this work, a report indicated that lack of GSDMD in mice prevented the onset and progression of Familial Mediterranean Fever, a disease in which aberrant pyrin inflammasome activities caused IL-1β oversecretion and pyroptosis [33]. Deficiency in GSDMD also protected mice against endotoxic shock, consistent with activation of this protein by intracellular LPS [14, 16]. A recent paper suggested an interplay between caspase-8 and caspase-11-GSDMD axis in the execution of endotoxic shock [34]. Collectively, these findings indicate that inactivation of GSDMD arrests pathogenic signals induced by various inflammasomes.

### Systemic inflammation and organ damage in NOMID mice are prevented by ablation of *Gsdmd*

**Fig 3:**
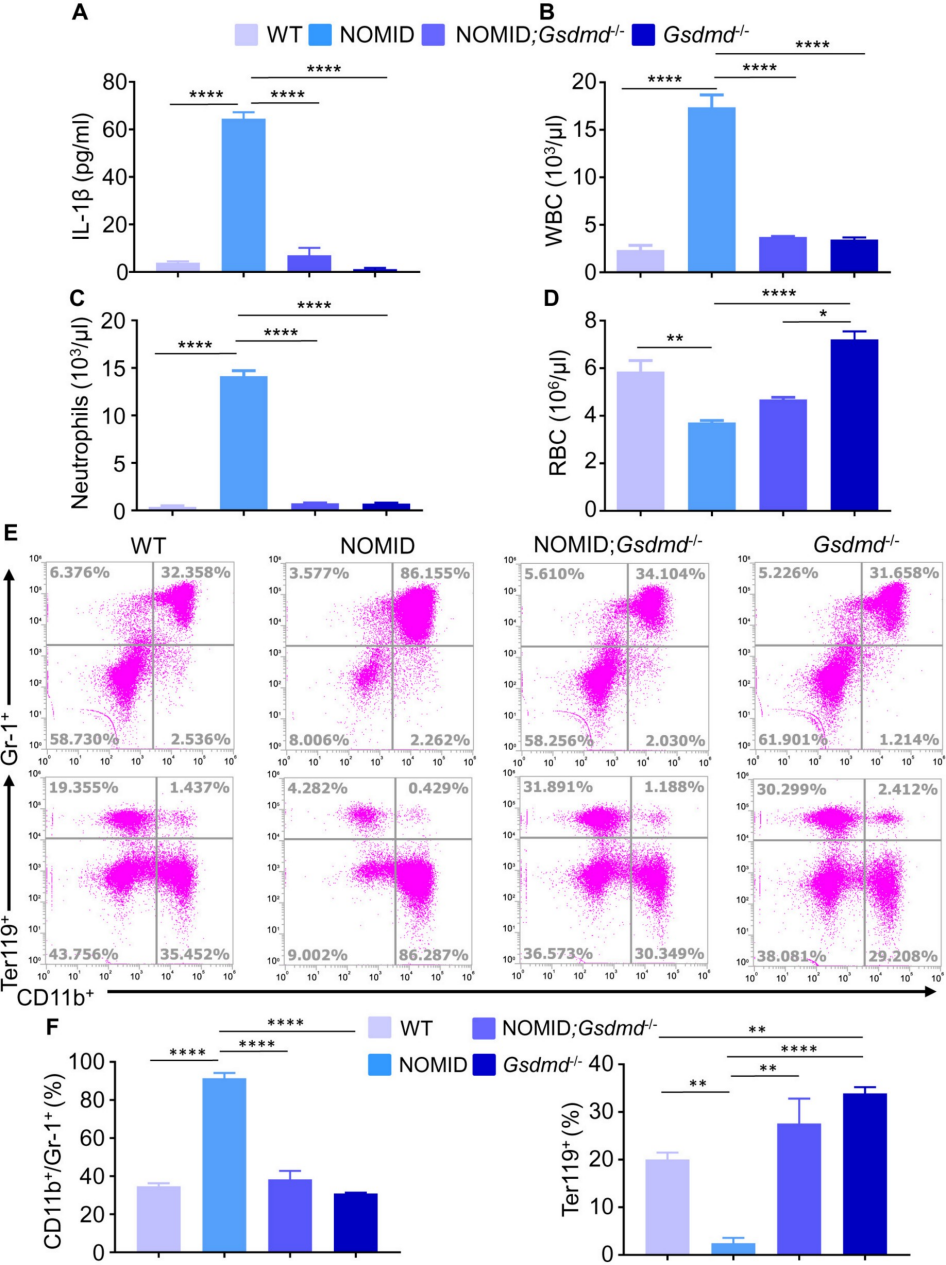
GSDMD deficiency prevents the onset of systemic inflammation in NOMID mice. (A) IL-1β levels in mouse bone marrow supernatants. (B) WBC counts. (C) Neutrophils. (D) RBCs. (E) Flow cytometry analysis of bone marrow cells stained with antibodies against CD11b, Gr-1, or Ter119. (F) Quantitative data from flow cytometry dot plots shown in panel E. Data are mean ± SEM. Three-week-old mice were used for the studies. WT mice (panel A: 2 males and 1 female; panel B and C: 3 males and 2 females; panel D: 5 males and 4 females; panel F: 2 males and 1 female for CD11b^+^/Gr-1^+^, and 3 males and 1 female for Ter119^+^). NOMID mice (panel A: 2 males and 1 female; panel B and C: 4 males and 3 females; panel D: 4 males and 3 females; panel F: 2 males and 1 female). NOMID;*Gsdmd*^−/−^ mice (panel A: 2 males and 1 female; panel B–F: 2 males and 1 female); *Gsdmd*^−/−^ mice: (panel A: 2 males and 1 female; panel B and C: 3 males and 3 females; panel D: 4 males and 4 females; panel F: 3 males and 1 female). The numerical values underlying Fig 3A, 3B, 3C, 3D and 3F can be found in S1 Data. *P < 0.05; **P < 0.005; ***P < 0.0005; ****P < 0.0001. GSDMD, gasdermin D; IL-1β, interleukin-1β; NOMID, neonatal-onset multisystem inflammatory disease; RBC, red blood cell; WBC, white blood cell; WT, wild-type.

IL-1β propagates inflammation through various mechanisms, including perturbation of chemokine and cytokine signaling networks, responses that lead to the expansion and recruitment of neutrophils to several organs. This cytokine also promotes anemia owing to its negative effects on erythroid progenitors and erythropoietin signaling as well as alteration of the expression of ferritin and ferroportin [35–38]. Accordingly, NOMID mice produced higher levels of IL-1β in bone marrow compared to WT counterparts (Fig 3A and S1 Data), a response that correlated with excessive GSDMD processing in vivo in bone marrow, though the cleaved fragment was barely detected in this compartment (S1B Fig). This observation was not unexpected considering that excessive generation of GSDMD-N caused cytolysis; as a result, the cleaved fragment may have been lost during the sampling process. NOMID mice also exhibited peripheral leukocytosis (Fig 3B and S1 Data) driven by neutrophilia (Fig 3C and S1 Data) and anemia (Fig 3D; S1C Fig and S1 Data), as we previously reported [25, 32]. The identity of myeloid cell subpopulations, which are prone to pyroptosis and propagate inflammation, in this model is unknown, a knowledge gap that future studies should address. In any case, while *Gsdmd* ablation had no effect on the number of blood cells compared to WT mice, it abrogated or attenuated the onset of leukocytosis and anemia in NOMID mice (Fig 3A–3D). Accordingly, the bone marrow compartment of NOMID mice contained abnormally high levels of Gr1^+^/CD11b^+^ cells and low levels of Ter119^+^ cells (Fig 3E and 3F; S1 Data), responses that were normalized upon *Gsdmd* deletion.

**Fig 4:**
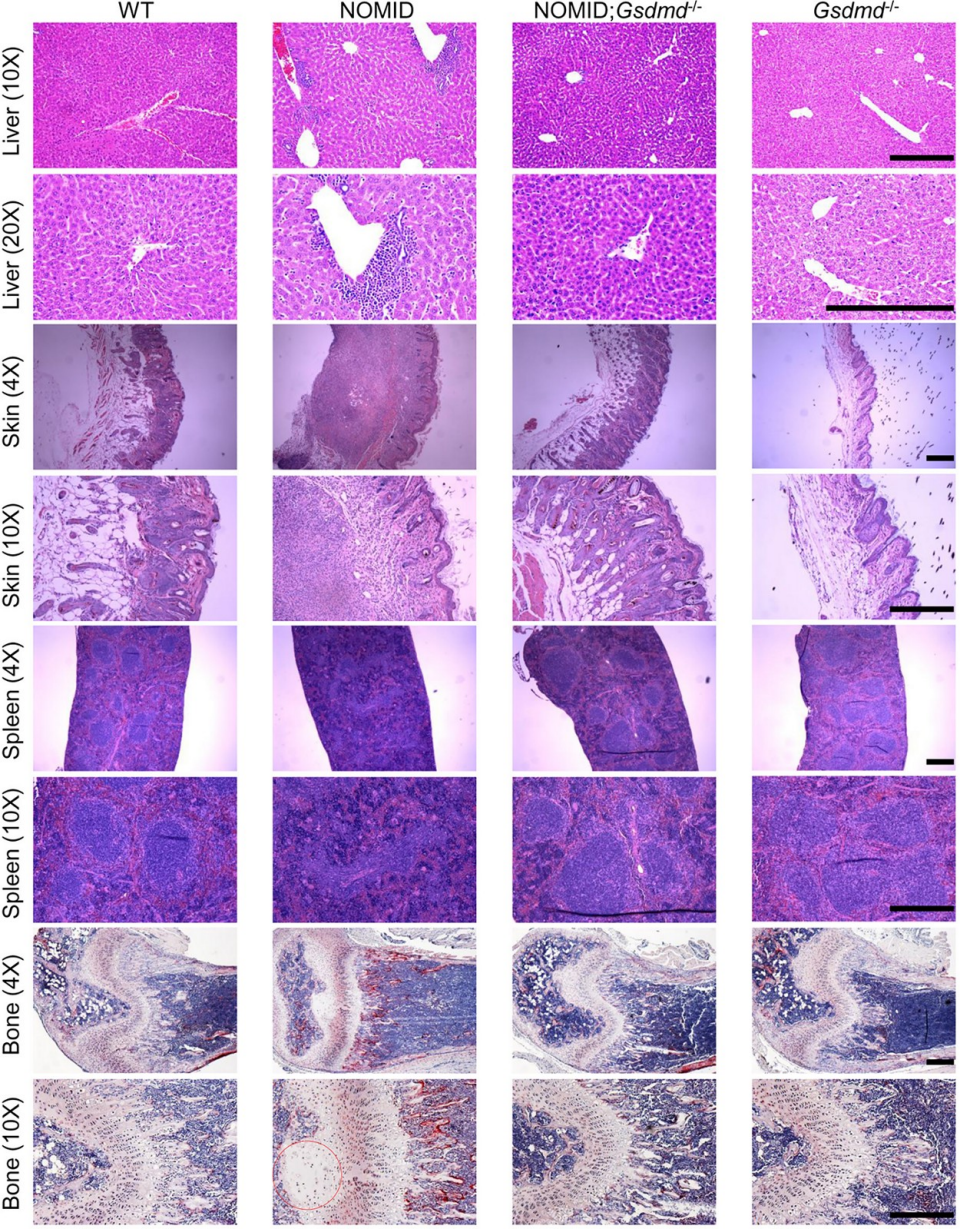
GSDMD deficiency prevents damage to multiple tissues in NOMID mice. Representative images of liver, skin, and spleen from 3-week-old WT mice (3 males and 1 female), NOMID mice (3 males and 1 female), NOMID;*Gsdmd*^*−/−*^ mice (2 males) and *Gsdmd*^*−/−*^ mice (3 males and 1 female) stained with HE. Femurs were stained for TRAP activity. Red circle indicates area of hypocellularity in the epiphysis. Osteoclasts are stained in red. Scale bar: 200 μm. GSDMD, gasdermin D; HE, hematoxylin–eosin; NOMID, neonatal-onset multisystem inflammatory disease; TRAP, tartrate-resistant acid phosphatase; WT, wild-type.

Histological analyses showed massive neutrophilic infiltration in the liver, dermal and hypodermal layers of the skin, and the spleen of NOMID mice compared to WT or *Gsdmd*^−/−^ counterparts (Fig 4). Inflammation in the spleen was characterized by disorganized structures of white and red pulps. Because skeletal complications—including low bone mass—are hallmarks of NOMID, we investigated these outcomes in NOMID mice. Histological examinations of skeletal elements showed disorganized columns of chondrocytes with profoundly altered morphology. The epiphysis was hypocellular (Fig 4), a phenotype that was previously reported to be caused by massive chondrocyte death [25, 32] and reminiscent of the human disease [39]. The number of osteoclasts, cells responsible for bone resorption, was markedly increased in NOMID mice relative to control mice. Remarkably, all organs that were analyzed in NOMID;*Gsdmd*^−/−^ mice were all spared from inflammation-induced damage (Fig 4). Thus, deletion of GSDMD abolishes inflammatory responses and organ demise in NOMID mice.

Blockade of IL-1 activity has been the main strategy for neutralizing pathogenic signals induced by this cytokine in CAPS and other autoinflammatory disorders. However, these drugs have shortcomings, including high cost and the requirement for parenteral administration. Thus, there is still a medical need for the development of safe and affordable drugs for the treatment of autoinflammatory diseases. Breakthrough research demonstrating that GSDMD-mediated pyroptosis releases cytoplasmic contents, including IL-1β and IL-18, offers a novel node for therapeutic intervention. Stemming from its mechanisms of action, blockade of GSDMD and subsequently pyroptosis should, in theory, provide superior efficacy compared with targeted blockade of IL-1β. The compelling evidence indicating that inactivation of GSDMD blocks inflammatory responses induced by the NLRP3 inflammasome lends support to discovery efforts aimed at identifying selective inhibitors of GSDMD actions.

## Materials and methods

### Ethics statement

All procedures were approved by the Institutional Animal Care and Use Committee (IACUC) of Washington University School of Medicine in St. Louis, Missouri. All experiments were performed in accordance with the relevant guidelines and regulations described in the IACUC-approved protocol number 20160245.

### Mice

*Gsdmd*^−/−^ mice [16] were kindly provided by Dr. V. M. Dixit (Genentech, South San Francisco, CA). *Nlrp3*^*−/−*^, *Casp1*^*−/−*^, and *Nlrp3*^*fl(D301N)/+*^ mice and *lysozyme M (LysM)-Cre* mice have previously been described [25, 31, 40, 41]. *Casp11*^*−/−*^ mice were purchased from The Jackson Laboratory. All mice were on the C57BL6J background, and mouse genotyping was performed by PCR.

### Peripheral blood and bone marrow analyses

Complete blood counts were performed by the Washington University School of Medicine DCM Diagnostic Laboratory as previously described [31]. Bone marrow cells were flushed out as previously described, and photographed [31].

### Cell cultures

BMMs were obtained by culturing mouse bone marrow cells in culture media containing a 1:25 dilution of supernatant from the fibroblastic cell line CMG 14–12 as a source of M-CSF, a mitogenic factor for BMMs, for approximately 5 days in a 10-cm dish following the procedures that we published [42]. Nonadherent cells were removed by vigorous washes with PBS, and adherent BMMs were detached with trypsin-EDTA and were cultured in culture media containing a 1:50 dilution of CMG at 4 × 10^4^/well in a 96-wells plate (for the analysis of IL-1β and LDH) or 1.2 × 10^6^/well in a 6-wells plate (for Western blot analysis).

### Western blot

BMMs were treated with 100 ng/mL LPS for 3 hours, then with 15 μM nigericin for 30 minutes. Extracts from BMMs or bone marrow cells were prepared by lysing cells or cell pellets, respectively, with RIPA buffer (50 mM Tris, 150 mM NaCl, 1 mM EDTA, 0.5% NaDOAc, 0.1% SDS, and 1.0% NP-40) plus phosphatase inhibitors and Complete Protease Inhibitor Cocktail (Roche, Brighton, MA). Protein concentrations were determined by the Bio-Rad method, and equal amounts of proteins were subjected to SDS-PAGE gels (12%). Proteins were transferred onto nitrocellulose membranes and incubated with GSDMD antibody (1:1,000, ab209845, Abcam, Cambridge, MA) or β-actin (1:5,000, sc-47778, Santa Cruz Biotechnology, Dallas, Texas) overnight at 4°C, followed by a 1-hour incubation with secondary goat anti-mouse IgG (1:5,000, A21058, Thermo Fisher Scientific, Grand Island, NY) or goat anti-rabbit IgG (1:5,000, A21109, Thermo Fisher Scientific), respectively. The results were visualized using Li-Cor Odyssey Infrared Imaging System (LI-COR Biosciences, Lincoln, Nebraska).

### Flow cytometry

Mouse bone marrow cells were flushed from femur and tibia. For flow cytometry analysis of the leukocytes, red blood cells (RBCs) were depleted with RBC lysis buffer (Roche, Brighton, MA). Cells (0.5–1 × 10^6^) were incubated with Fc block (anti-mouse CD16/32, BioLegend, San Diego, CA) to block nonspecific Fc binding, stained with isotype control or APC-anti-mouse Ter119 (BioLegend, San Diego, CA), FITC-anti-mouse CD11b (eBioscience, Grand Island, NY), and PE-anti-mouse Ly-6G/Ly-6C (Gr1) antibody (BioLegend, San Diego, CA) according to the supplier’s instructions. Flow cytometry was performed using BD LSRFortessa or BD FACSCanto II Flow Cytometer system, followed by analysis with FlowJo software (Tree Star, Ashland, Oregon).

### Histology

All tissues were harvested and fixed in 10% formalin. Long bones were decalcified in 14% (w/v) EDTA for 5 days at room temperature. All tissues were embedded in paraffin, sectioned at 5 μm thickness, and mounted on glass slides. Sections were stained with hematoxylin–eosin (HE) or TRAP as previously described [42].

### Cytotoxicity assay and IL-1β ELISA

BMMs were treated with 100 ng/mL LPS for 3 hours, then with 15 μM nigericin for 30 minutes.

Cell death was assessed by the release of LDH using LDH Cytotoxicity Detection Kit (TaKaRa, Mountain View, CA). IL-1β levels in conditioned media were measured by ELISA (eBiosciences, Grand Island, NY). For IL-1β measurements in bone marrow, flushed bone marrow was centrifuged, and the supernatants were collected as described previously [42]. IL-1β levels were quantified using the eBioscience ELISA kit.

### Statistical analysis

Statistical analysis was performed using one-way ANOVA with Tukey's multiple comparisons test or two-way ANOVA with Tukey's multiple comparisons test in GraphPad Prism 7.

## Supporting information

S1 Fig GSDMD deficiency prevents the stunt and anemic phenotype of NOMID mice. (A) Representative pictures of mice from each genotype from a cohort of mice different from the one shown in Fig 2. Red arrows indicate skin lesions. Pictures of 12-day-old WT mice (2 males); NOMID mice (1 male and 1 female), NOMID;*Gsdmd*^*−/−*^ mice (2 males), and *Gsdmd*^*−/−*^ mice (1 male and 1 female). (B) Western blot analysis of bone marrow cell extracts. (C) Pictures of pellets of bone marrow cells isolated from 3-week-old WT, NOMID, NOMID;*Gsdmd*^−/−^, or *Gsdmd*^−/−^ mice.
(PDF)

S1 Data Data for Fig 1B, 1C, 1E and 1F, Fig 2B and 2D, and Fig 3A, 3B, 3C, 3D and 3F. (XLSX)

## Abbreviations

ASC: apoptosis-associated speck-like protein containing a CARD
BMM: bone marrow–derived macrophage
CAPS: cryopyrin-associated periodic syndrome
FCAS: familial cold autoinflammatory syndrome
GSDMD: gasdermin D
GSDMD-C: GSDMD C-terminal domain
GSDMD-N: GSDMD N-terminal domain
HE: hematoxylin–eosin
IACUC: Institutional Animal Care and Use Committee
IL-1β: interleukin-1β
LDH: lactate dehydrogenase
LPS: lipopolysaccharide
M-CSF: macrophage colony-stimulating factor
MWS: Muckle-Wells syndrome
NOMID: neonatal-onset multisystem inflammatory disease
TRAP: tartrate-resistant acid phosphatase
WT: wild-type
