# Supplementary material for: Gasdermin D mediates the pathogenesis of neonatal-onset multisystem inflammatory disease in mice
Source: PLoS Biol. 2018 Nov 2;16(11):e3000047. doi: 10.1371/journal.pbio.3000047 (PMC6235378; doi:10.1371/journal.pbio.3000047)
Supplement: S1 Fig — (A) Representative pictures of mice from each genotype from a cohort of mice different from the one shown in Fig 2. Red arrows indicate skin lesions. Pictures of 12-day-old WT mice (2 males); NOMID mice (1 male and 1 female), NOMID;Gsdmd−/− mice (2 males), and Gsdmd−/− mice (1 male and 1 female). (B) Western blot analysis of bone marrow cell extracts. (C) Pictures of pellets of bone marrow cells isolated from 3-week-old WT, NOMID, NOMID;Gsdmd−/−, or Gsdmd−/− mice. (PDF) [file pbio.3000047.s001.pdf]

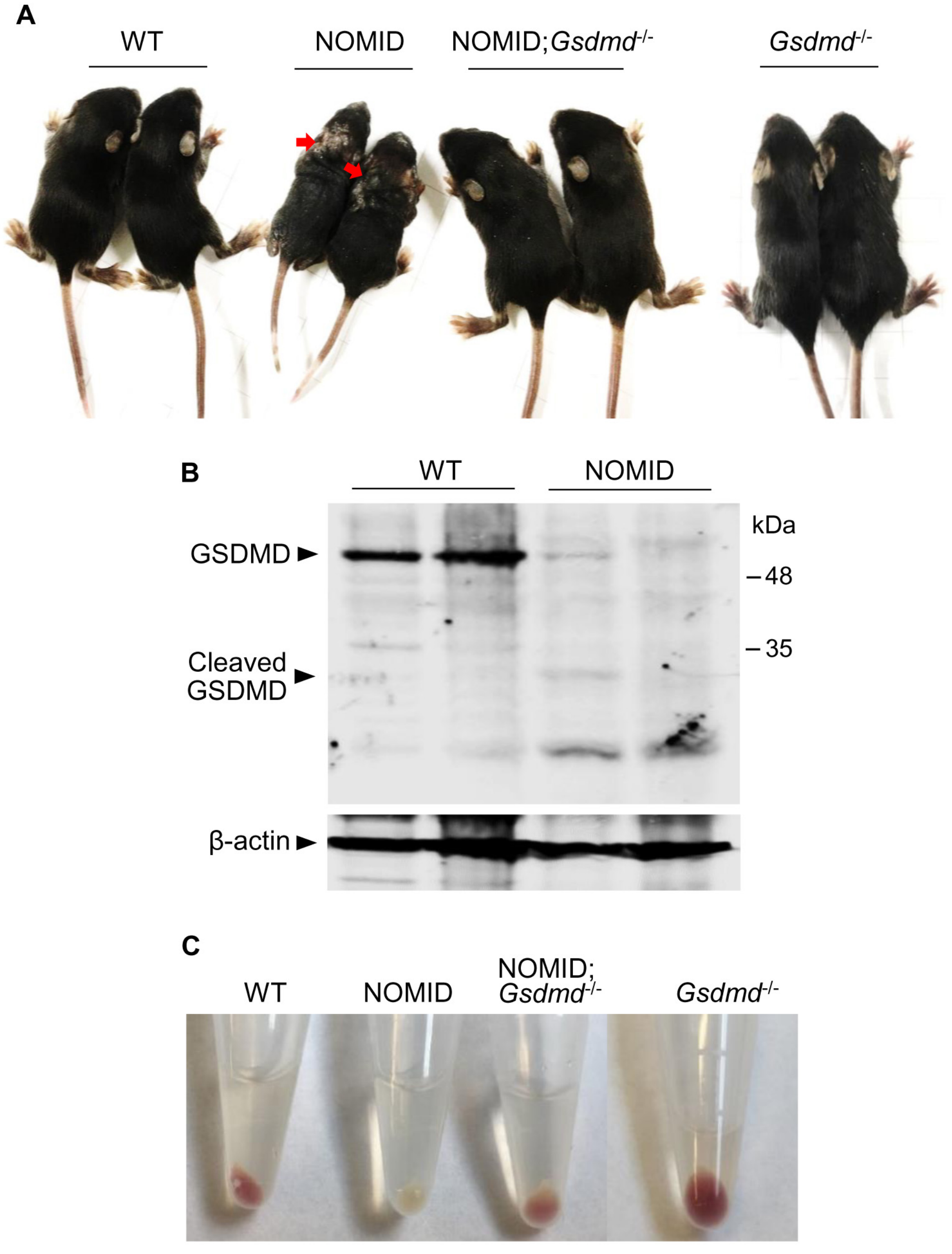

A
WT
NOMID
NOMID;*Gsdmd*-/-
Gsdmd-/-
B
WT
NOMID
kDa
GSDMD
48
35
Cleaved GSDMD
β-actin
C
WT
NOMID
NOMID; *Gsdmd*-/-
Gsdmd-/-
